# Effects of aperture on Marchenko focussing functions and their radiation behaviour at depth

**DOI:** 10.1111/1365-2478.12735

**Published:** 2019-01-11

**Authors:** Yanadet Sripanich, Ivan Vasconcelos

**Affiliations:** ^1^ Department of Earth Sciences Utrecht University P.O. Box 80115 8305 TC Utrecht The Netherlands

**Keywords:** Virtual source, Focussing function, Time imaging

## Abstract

A focussing function is a specially constructed field that focusses on to a purely downgoing pulse at a specified subsurface position upon injection into the medium. Such focussing functions are key ingredients in the Marchenko method and in its applications such as retrieving Green's functions, redatuming, imaging with multiples and synthesizing the response of virtual sources/receiver arrays at depth. In this study, we show how the focussing function and its corresponding focussed response at a specified subsurface position are heavily influenced by the aperture of the source/receiver array at the surface. We describe such effects by considering focussing functions in the context of time‐domain imaging, offering explicit connections between time processing and Marchenko focussing. In particular, we show that the focussed response radiates in the direction perpendicular to the line drawn from the centre of the surface data array aperture to the focussed position in the time‐imaging domain, that is, in time‐migration coordinates. The corresponding direction in the Cartesian domain follows from the sum (superposition) of the time‐domain direction and the directional change due to time‐to‐depth conversion. Therefore, the result from this study provides a better understanding of focussing functions and has implications in applications such as the construction of amplitude‐preserving redatuming and imaging, where the directional dependence of the focussed response plays a key role in controlling amplitude distortions.

## 1 INTRODUCTION

**Figure 1:**
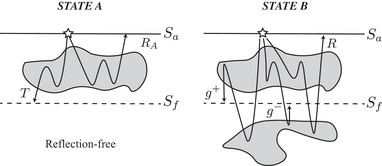
The two acoustic states A and B used in the derivation of the single‐sided Green's function representations (equation (1)). State A has a reflection‐free region (e.g. homogeneous) below a specified surface $S_{f}$ that contains the focussing points at depth. The reflection and transmission responses (operators) in this state are $R_{A}$ and *T*, respectively. State B is the true medium, which is the same medium of State A between $S_{a}$ and $S_{f}$, but below $S_{f}$ it is arbitrarily inhomogeneous. The total reflection response in this state is *R*, usually assumed to be represented by the recorded reflection data on the surface $S_{a}$, $g^{+}$ and $g^{-}$ are, respectively, the desired downgoing and upgoing Green's functions for virtual receivers on $S_{f}$. These can be obtained by solving the time‐constrained coupled Marchenko equations based on an initial focussing function from a smooth (migration) velocity model.

The Marchenko method is a relatively novel technique that relates surface reflection data to Green's function from any subsurface position based on the concept of focussing function. The theoretical construction of focussing functions is generally done using the one‐way reciprocity theorems and two specific acoustic states – the true medium and its corresponding truncated medium with a homogeneous half space below the chosen focussing depth as shown in Fig. 1 (Slob *et al*. 2014; Wapenaar *et al*. 2014a,b; van der Neut, Vasconcelos and Wapenaar 2015). By definition, the Marchenko focussing function is related to the inverse transmission operator between the surface and the chosen focussing location at depth in the truncated medium. The truncated medium itself can be arbitrarily heterogeneous, and free‐surface effects can also be included in the construction of focussing functions (Ravasi 2017; Singh *et al*. 2017). Upon propagation (injection) of focussing functions into the medium under investigation, the wavefields will interact with the medium in such a way that leads to a purely downgoing, band‐limited delta function (e.g. Wapenaar *et al*. 2014b) at the specified subsurface position – hence the term *focussing*.

**Figure 2:**
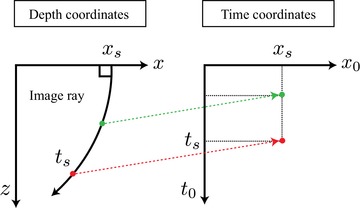
The relationship between the time‐ and depth‐imaging domains defined by image rays. An example image ray that originates from $x_{s}$ with slowness vector normal to the surface travels along a curvilinear path in spatial Cartesian coordinates (x,z). Every point along this raypath is mapped to the time‐imaging domain or image‐ray coordinates ($x_{0},t_{0}$) according to the traveltime $t_{s}$ along the ray.

In conventional seismic imaging, a subsurface model can be expressed in either the time‐ or depth‐imaging domain (Yilmaz 2001). The former comes from time‐domain processing, which is computationally efficient, but has limited applicability in complex media. On the other hand, the latter is the result of depth‐domain processing, which offers a better performance when dealing with complex subsurface, but comes at the expense of considerably higher computational cost. These two domains are related via the image‐ray mapping (Fig. 2) that defines the time‐to‐depth conversion process (Hubral 1977; Yilmaz 2001; Cameron, Fomel and Sethian 2007; Sripanich and Fomel 2018). When the medium in question is moderately (laterally) complex and the assumptions behind time‐domain processing are valid, its subsurface models expressed in either the time‐ or depth‐imaging domain properly represent the same, actual medium. Thus, the position of the focussed responses in both time‐ and depth‐imaging domain correctly map to one another through the image rays.

In this study, under the regime of time‐domain imaging and its assumptions, we show that the focussed responses in the time‐ and depth‐imaging domains are not only related in terms of spatial locations, but also amplitudes. Due to the assumptions of effective medium (i.e. time migration) velocity and straight‐ray geometry in time imaging, we first show that there is a straightforward relationship between the radiation direction of the focussed response in the time‐imaging domain and the aperture of the source/receiver array at the surface. The corresponding radiation direction in the depth‐imaging domain can be obtained after taking into account the additional change in direction from the time‐to‐depth conversion process. This knowledge may lead to key implications in the design of directionally controlled, amplitude‐preserving virtual subsurface sources (i.e. redatuming) based on the concept of Marchenko focussing.

## 2 A BRIEF OVERVIEW OF FOCUSSING FUNCTIONS

In the Marchenko method, the following single‐sided Green's function representations (Wapenaar *et al*. 2014a,b; van der Neut *et al*. 2015) can be established from one‐way reciprocity theorems (Wapenaar and Grimbergen 1996):

(1) $$\begin{array}{ccc} g^{-}\left(x_{f},x_{a},t\right) & = & \int_{S_{a}}R\left(x_{a}^{\prime },x_{a},t\right)*f_{1}^{+}\left(x_{a}^{\prime },x_{f},t\right)dx_{a}^{\prime }\\ & & -f_{1}^{-}\left(x_{a},x_{f},t\right),\\ -g^{+}\left(x_{f},x_{a},t\right) & = & \int_{S_{a}}R\left(x_{a}^{\prime },x_{a},t\right)*f_{1}^{-}\left(x_{a}^{\prime },x_{f},-t\right)dx_{a}^{\prime }\\ & & -f_{1}^{+}\left(x_{a},x_{f},-t\right), \end{array}$$

where * denotes time convolution. $x_{a}$ and $x_{f}$ denote the lateral position on the acquisition surface $S_{a}$ and $S_{f}$, respectively. Using this system of equations, the downgoing ($g^{+}$) and upgoing ($g^{-}$) Green's functions at locations on the focussing surface $S_{f}$ can be found from the knowledge of the reflection operator *R*, together with the downgoing ($f_{1}^{+}$) and upgoing ($f_{1}^{-}$) focussing functions. Figure 1 illustrates the two states used to derive the single‐sided representations in equation (1).

**Figure 3:**
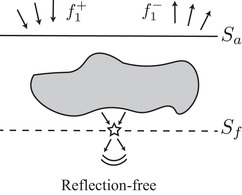
A schematic illustrating the downgoing focussing function $f_{1}^{+}$ and its reflection $f_{1}^{-}$ in the truncated medium (state A).

The downgoing focussing function $f_{1}^{+}$ (Fig. 3) is defined as the inverse of transmission operator *T* in state A (Fig. 1), which can be written as (Wapenaar *et al*. 2014b, 2017):

(2) $$\delta \left(x-x_{f}\right)\delta \left(t\right)=\int_{S_{a}}T\left(x_{f},x_{a}^{\prime },t\right)*f_{1}^{+}\left(x_{a}^{\prime },x_{f},t\right)\;dx_{a}^{\prime }.$$

Note that $\delta (x-x_{f})$ is a delta function on the focussing surface $S_{f}$. In the Fourier domain at some angular frequency ω, equation (2) can be expressed as (van der Neut *et al*. 2015; Vasconcelos *et al*. 2015):

(3) $$\delta \left(x-x_{f}\right)=\int_{S_{a}}\hat{T}\left(x_{f},x_{a}^{\prime },\omega \right){\hat{f}}_{1}^{+}\left(x_{a}^{\prime },x_{f},\omega \right)\;dx_{a}^{\prime },$$

or equivalently, when numerically discretizing the integral, in matrix notation:

(4) $$I=\hat{T}{\hat{F}}_{1}^{+}.$$

$\hat{T}$ denotes a $n_{f}\times n_{a}$ transmission matrix for the truncated medium with elements representing Green's functions between $n_{a}$ points on the acquisition surface and $n_{f}$ focussed points at some specified depth. We seek ${\hat{F}}_{1}^{+}$ denoting a $n_{a}\times n_{f}$ downward‐going focussing function that is an inverse of $\hat{T}$. When the medium is smooth, there is only one event in $f_{1}^{+}$ associated with the direct waves traveling from the specified focussing position to the surface $S_{a}$. On the other hand, if the medium produces scattering (i.e. reflections, diffractions), parts of the signals are reflected upward when injecting the downgoing focussing function $f_{1}^{+}$ to generate a focussed field at some specified location. This upward reflected response is referred to as $f_{1}^{-}$ (Fig. 3) and the combination of the two functions gives the total focussing function:

(5) $$f_{1}\left(x_{a},x_{f},t\right)=f_{1}^{+}\left(x_{a},x_{f},t\right)+f_{1}^{-}\left(x_{a},x_{f},t\right).$$

A good illustration of this concept can be found in Figs. 3 and 4 of Wapenaar *et al*. (2014b) and in Figs. 7 and 8 of Wapenaar *et al*. (2017).

**Figure 4:**
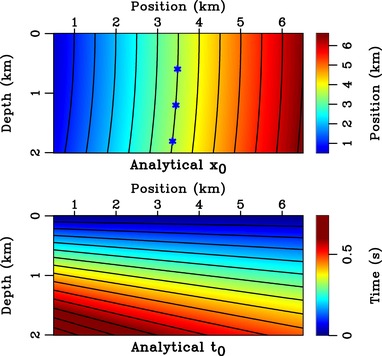
Analytical *x*_0_ with its contours overlain, denoting image rays, and analytical *t*_0_ with its contours denoting image wavefronts. The stars denote three scatterer locations along the image ray that emerges at 3.5 km on the surface of the model.

To create a focussed field in the subsurface, Wapenaar *et al*. (2014b) show that one needs to inject the total downgoing field *f* at $S_{a}$ given by

(6) $$f\left(x_{f},x_{a},t\right)=f_{1}^{+}\left(x_{a},x_{f},t\right)-f_{1}^{-}\left(x_{a},x_{f},-t\right),$$

where we swap the position of the arguments $x_{f}$ and $x_{a}$ to emphasize that *f* is to be injected from $S_{a}$ (Wapenaar *et al*. 2014b; Thorbecke *et al*. 2017). The $f_{1}^{-}$ is now time‐reversed and subtracted from $f_{1}^{+}$ to handle, in real time, the unwanted reflections resulting from the injection of $f_{1}^{+}$. Equation (1) can then be rewritten using *f* as follows (Wapenaar *et al*. 2017):

(7) $$\begin{array}{ccc} & & g\left(x_{a},x_{f},t\right)=g\left(x_{f},x_{a},t\right)\\ & & \;=\int_{S_{a}}R\left(x_{a}^{\prime },x_{a},t\right)*f\left(x_{f},x_{a}^{\prime },t\right)dx_{a}^{\prime }+f\left(x_{f},x_{a},-t\right), \end{array}$$

where the first equality is due to the source–receiver reciprocity of Green's function $g=g^{+}+g^{-}$. Therefore, at the surface $S_{a}$, the total field is a summation of the incident field $f(x_{f},x_{a},t)$ and its reflected response $g\left(x_{f},x_{a},t\right)-f\left(x_{f},x_{a},-t\right)$. The homogeneous Green's function $g_{h}$ from the virtual source at the specified focussing position to any point in the medium can then be obtained by summing of the total field at $S_{a}$ with a time‐reversed version of itself. This result is originally presented by Broggini and Snieder (2012) who validate it in 1D media. Extensions to 2D and 3D media can be found in, for example Wapenaar *et al*. (2017). Mathematically, this amounts to

(8) $$\begin{array}{ccc} (f\left(x_{f},x_{a},t\right) & & +\;g\left(x_{a},x_{f},t\right)-f\left(x_{f},x_{a},-t\right))+(f\left(x_{f},x_{a},-t\right)\\ & & +\;g\left(x_{a},x_{f},-t\right)-f\left(x_{f},x_{a},t\right)), \end{array}$$

which gives the homogeneous Green's function $g_{h}\left(x_{f},x_{a},t\right)$ defined as

(9) $$g_{h}\left(x_{a},x_{f},t\right)=g\left(x_{a},x_{f},t\right)+g\left(x_{a},x_{f},-t\right).$$

In the following sections, we will specifically rely on the focussing function *f* (equation (6)) and the resulting homogeneous Green's function $g_{h}$ from the virtual source (equation (9)) to establish the connection between the radiating direction of the focussed response (i.e. the virtual source) in the time‐ and depth‐imaging domains. We follow the workflow as described by Wapenaar *et al*. (2014b), van der Neut *et al*. (2015) and Thorbecke *et al*. (2017) to solve for the focussing functions *f*. This consists of three main steps:

1. approximate the initial focussing function $f_{1,0}^{+}$ by the time‐reversed direct‐wave Green's function (e.g. from a smooth/background model);
2. using the known *R*‐operator and initial $f_{1,0}^{+}$, solve the time‐windowed Green's function representations (coupled Marchenko equations) by iterative substitution (van der Neut *et al*. 2015) for $f_{1}^{-}$ and an update of $f_{1,m}^{+}$, where $f_{1}^{+}=f_{1,0}^{+}+f_{1,m}^{+}$;
3. finally, use the estimates of $f_{1}^{+}$ and $f_{1}^{-}$ from the previous step to estimate the homogeneous Green's functions, using equations (6), (7) and (9).

In this study, we consider the effects of the surface source/receiver array aperture on the radiation direction at depth. We achieve this in Step 1 by windowing (multiplying by a Heaviside weight) *R* and $f_{1,0}^{+}$ corresponding to different source/receiver array sizes. Implementing Steps 2 and 3 then gives estimates of focussing functions, which will be injected into the medium for our study on the radiation direction of the resulting homogeneous Green's function. Because we directly modify the input *R* and $f_{1,0}^{+}$, it is to be expected that the focussing functions obtained this way for different surface source/receiver array apertures are not the same as those obtained from simply windowing the focussing functions with a larger source/receiver array aperture as we shall see later in our numerical examples.

## 3 FOCUSSING IN TIME‐ AND DEPTH‐IMAGING DOMAINS

Upon the retrieval of focussing functions *f* (equation (6)), we employ the acoustic wave equation and its counterpart in the time‐imaging domain (Fomel 2013) to back‐propagate them into the subsurface model. Their expressions can be given as follows.

- **Depth (Cartesian) coordinates**
  (10) $$\frac{\partial^{2}u}{\partial x^{2}}+\frac{\partial^{2}u}{\partial z^{2}}=\frac{1}{v^{2}\left(x,z\right)}\frac{\partial^{2}u}{\partial t^{2}}.$$
- **Time (image‐ray) coordinates**
  (11) $$v_{d}^{2}\left(x_{0},t_{0}\right)\frac{\partial^{2}u}{\partial x_{0}^{2}}+\frac{\partial^{2}u}{\partial t_{0}^{2}}=\frac{\partial^{2}u}{\partial t^{2}}.$$

We use *u* to denote the wavefield, *v* is the medium velocity in the Cartesian coordinates, and $v_{d}$ is the Dix velocity obtained after applying the Dix inversion on time‐migration velocity (e.g. Cameron *et al*. 2007; Li and Fomel 2015; Sripanich and Fomel 2018). The Dix velocity $v_{d}$ lives in the image‐ray coordinates defined by *x*_0_ and *t*_0_ for the surface escape location and the one‐way traveltime of image rays, respectively. In the next section, we use equations (10) and (11) to back‐propagate the focussing function injected at the surface, and establish connections between the directionally dependent properties of the focussed responses in both domains.

## 4 RADIATION DIRECTION AND DATA APERTURE

### 4.1 Direct waves

We first study the focussed responses from the focussing functions corresponding to direct waves only (Thorbecke 1997). This experiment represents the case of smooth‐background media with $f_{1}^{-}=0$ and the initial $f_{1}^{+}$ approximated by the time‐reversed direct‐wave Green's function, which is taken as a leading‐order estimate of the time‐reversed transmission response. Using the notation from equation (4), we can write this as

(12) $${\hat{F}}_{1,0}^{+}\approx {\hat{T}}_{0}^{†},$$

where † denotes conjugate transpose (adjoint). Here, ${\hat{F}}_{1,0}^{+}$ is the focussing operator that contains the frequency‐domain version of the initial focussing functions $f_{1,0}^{+}$ between the surface $S_{a}$ and all discrete focussing locations on $S_{f}$ at depth. ${\hat{T}}_{0}$ is the discrete truncated‐medium transmission operator for a smooth‐background medium, containing only direct arrivals, unlike the full‐wavefield $\hat{T}$ that yields the true‐medium, full‐waveform ${\hat{F}}_{1}^{+}$ (equation (4)).

We consider a synthetic medium (Fig. 4) with constant velocity gradients in both *x* and *z* directions given by

**Figure 5:**
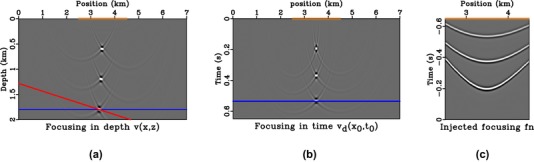
Direct‐wave focussing in depth (a) and time (b) domains, where orange boxes denote the surface aperture of the source/receiver array. We can observe that the focussed positions bend laterally, following the image‐ray direction in the depth domain, (a) but align along the vertical in the time domain (b). The blue line is parallel to the injecting‐source surface for reverse‐time propagation, and the red line is parallel to the image wavefront. The injected data, symmetric around the 3.5 km, is shown in (c).

(13) v(x,z)=2.0+0.6z+0.25x,

because it is convenient for our arguments as it has an analytical image‐ray map (Li and Fomel 2015; Sripanich and Fomel 2018). We consider three scatterers along the image rays originating from 3.5 km as noted by the stars in Fig. 4. According to the relationship between the two coordinates as defined by image rays (Fig. 2), we expect the focussed responses to align along the vertical in the time coordinates and follow the trajectory of the image ray in the depth coordinates. The result from injection of the focussing function with symmetric aperture (with respect to the central image ray at 3.5 km) from 2.5 to 4.5 km is shown in Fig. 5. The solid red line in Fig. 5 denotes the image wavefront, that is, a surface of constant *t*_0_, which is parallel to the focussed response in the depth domain in this case. On the other hand, the solid blue line is perpendicular to the line drawn from the middle of the source/receiver array (injecting sources for reverse‐time propagation) to the focussed position in the time domain. This blue line is observed to be parallel to the focussed response due to the straight‐ray geometry in time‐domain imaging. In this experiment, the blue line is horizontal because the receivers are distributed equally from 2.5 to 4.5 km with the middle at 3.5 km, corresponding to the scatterer focal locations in the time‐domain focussed fields.

**Figure 6:**
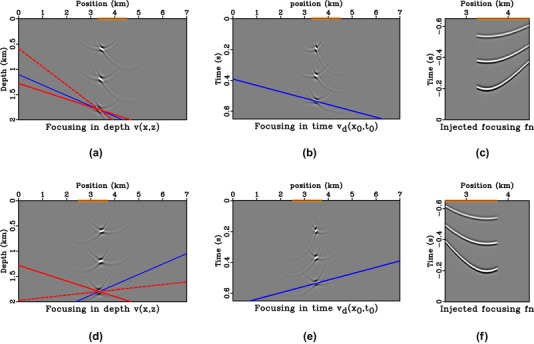
Direct‐wave focussing in depth (a,d) and time domains (b,e). The asymmetric source/receiver array is denoted by the orange boxes. The results from right‐skewed injection are shown in (a)–(c) and those from left‐skewed injection are shown in (d)–(f). The dashed red line has a cumulative slope of the solid red and blue lines.

To investigate aperture effects, we consider two receiver geometries in the 3.25–4 km and 2.5–3.75 km position ranges. Both are asymmetric with respect to the focal position of 3.5 km. The results from injection of *f* obtained with asymmetric aperture are shown in Fig. 6, where we note the additional dashed red line that has a combined slope of the solid red and blue lines. In other words, if the solid blue line (asymmetric aperture) has a slope $m_{b}$ and the solid red line (image ray bending) has a slope $m_{r}$, the dashed red line (final directionality) has a slope of $m_{b}+m_{r}$. In this study, we obtain $m_{b}$ from

(14) $$\begin{array}{c} m_{b}=\frac{\text{aperture}\;\text{centre}\;\text{--}\;\text{lateral}\;\text{focal}\;\text{position}\;\text{in}\;\text{time-imaging}\;\text{domain}}{\text{event}\;\text{one-way}\;\text{time}\times \text{migration}\;\text{velocity}}\;, \end{array}$$

and $m_{r}$ directly from the image‐ray map. Note that this calculation is done based on Heaviside weighting, where the aperture centre is at the middle of the source/receiver array. Moreover, both slopes must be scaled properly to handle different grid sizes in both time‐ and depth‐imaging domains before comparing with wavefields.

Considering the results from Figs. 5 and 6, we can make several observations.

1. Having a surface source/receiver array that is asymmetric relative to the focal position (i.e. limiting data aperture) is equivalent to windowing/weighting parts of the data, which changes the dynamics (amplitudes) of the resulting focussed field, but not the location of the focal points. Because the focussed positions remain the same, they are controlled solely by the kinematics (phase/traveltime) of the available data.
2. In the time‐imaging domain, the directionality of the focussed response (blue line) is perpendicular to the line drawn from the middle of the surface source/receiver array to the focussed position in the time (image‐ray) coordinates.
3. In depth (Cartesian) coordinates, the directionality of the focussed response is a direct combination (dashed red line) of the effects from limited aperture (blue) and the bending of image rays (red line) used for time‐to‐depth conversion. Specifically, the slope of dashed red line is the sum of those of the solid red and blue lines.

An interpretation of such observations can be achieved by considering a least‐squares estimate of $F_{1}^{+}$ based on equation (4) given by

(15) $${\left({\hat{F}}_{1}^{+}\right)}_{\text{LS}}={\left({\hat{T}}^{†}\hat{T}\right)}^{-1}{\hat{T}}^{†}=D{\hat{T}}^{†},$$

where D is a deblurring operator. In other words, a focussing function that will focus at a specified position can be interpreted as a deblurred version of the adjoint (time‐reversed) transmission response as used in this experiment (equation (12)). Because D is zero‐phase (being the inverse of the operator ${\hat{T}}^{†}\hat{T}$), the amplitude weight of the surface data (Heaviside weighting in our case) controls the directionality of the focussed response in both time and depth domains, while not altering the location of the focal point. A mathematically extensive treatment of directionally varying ‘amplitude blurring’, in the context of depth imaging, is presented by Thomson, Kitchenside and Fletcher (2016). In the following section, we build on the above remarks and extend our conclusions to the cases of focussing functions for 1D laterally homogeneous and 2D heterogeneous media.

### 4.2 Focussing functions with 1D model

**Figure 7:**
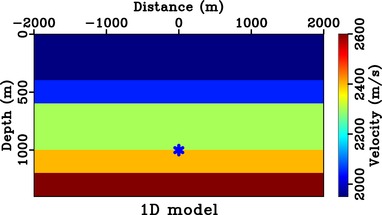
One‐dimensional synthetic velocity model with vertical image rays. The star denotes the considered focussing positions on the third reflector.

**Figure 8:**
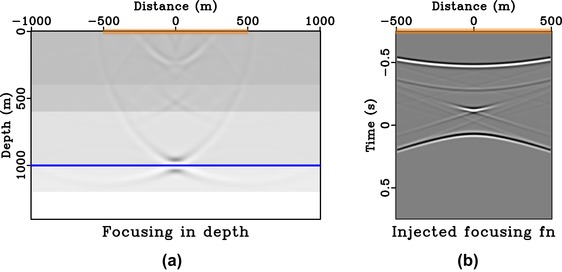
Focussing experiment in 1D model. Subpart (a) is the focussed response from injecting the focussing function shown in (b) that is obtained with symmetric aperture (orange box). In this example, the image rays are vertical and thus the directionality of the focussed response is represented by a horizontal solid blue line.

We consider the 1D model shown in Fig. 7 and a focussing point at (0, 1000 m) on the third reflector. The image rays are vertical in this model and the additional change of the radiation direction due to the time‐to‐depth conversion can thus be neglected. We follow the procedure outlined above and obtain the initial focussing function for the Marchenko method from the time‐reversed direct‐wave Green's function from this focussing position. Figure 8 shows the final estimate of the focussing function (*f*) obtained and injected with a symmetric surface aperture and its corresponding homogeneous Green's function at time equal to zero.

**Figure 9:**
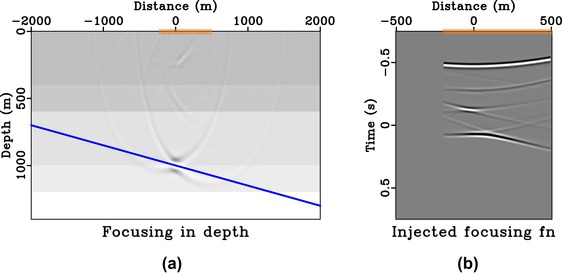
Focussing experiment in 1D model with asymmetric injection. Subpart (a) is the focussed response from the focussing function shown in (b) that is obtained with asymmetric aperture (orange box). The image rays are also vertical in this example, but the directionality of the focussed response is controlled by the effects of asymmetric injection (solid blue). Note that the waveforms in (b) are not equivalent to spatially windowing those of Fig. 8(b).

From Fig. 8, we can clearly see that the focussed response has a directionality defined by the solid blue line similarly to the case of direct waves (Fig. 5). Note that the remaining edge artefacts of the focussed response are due to the relatively small surface aperture (from −500 to 500 m) considered here. Similar results for the case of asymmetric surface aperture (from −200 to 500 m) are shown in Fig. 9. The focussed response shown in Fig. 9(a) now has a directionality defined by the solid blue line and a similar conclusion can be drawn as in the case of direct waves. We note that the resulting focussing function obtained in Fig. 9(b) is not the same as simply windowing the full focussing function with symmetric aperture in Fig. 8(b).

These results are not entirely surprising because, given the choice of a direct‐wave Green's function with an arbitrary spatially dependent weighting (e.g. limited aperture or deblurring‐derived weights), the Marchenko method (equation (1)) solves for $f_{1}^{+}$ and $f_{1}^{-}$ that in turn would lead to a focussed response that is directionally weighted according to such a choice. Therefore, the observations on the effects of aperture on the focussed response of direct waves studied in the previous section can be extended to the case of any version of any spatially weighted Marchenko focussing functions.

### 4.3 Focussing functions with 2D model

**Figure 10:**
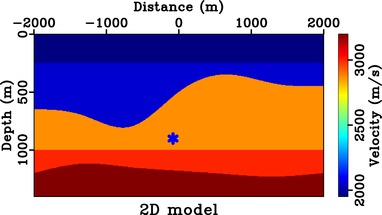
Two‐dimensional synthetic velocity model with bending image rays. The star denotes the considered focussing position at (−80, 900 m).

**Figure 11:**
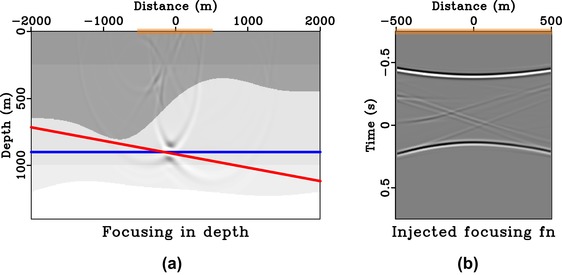
Focussing experiment in 2D model. Subpart (a) is the focussed response from injecting the focussing function shown in (b) that is obtained with symmetric aperture (orange box). The image rays are not vertical in this example and the directionality of the focussed response (dashed red) is controlled by a cumulative effect of symmetric injection (solid blue) and the image‐ray bending (solid red). Because the solid blue line is horizontal, the solid and dashed red lines are overlaying each other.

**Figure 12:**
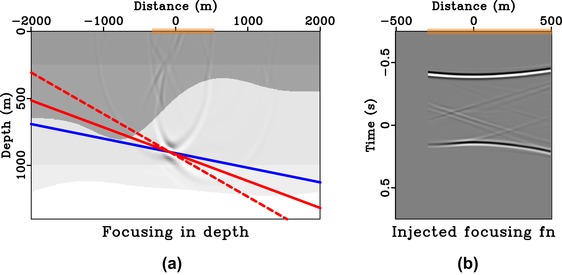
Focussing experiment in 2D model with asymmetric injection. Subpart (a) is the focussed response from injecting the focussing function shown in (b) that is obtained with asymmetric aperture (orange box). The image rays are not vertical in this example and the directionality of the focussed response (dashed red) is controlled by a cumulative effect of asymmetric injection (solid blue) and the image‐ray bending (solid red). Note that the waveforms in (b) are not equivalent to spatially windowing those in Fig. 12(b).

Next, we turn to a 2D laterally heterogeneous model with bending image rays (Fig. 10), where both effects from asymmetric surface aperture and the time‐to‐depth conversion are important. We consider the focussing position at (−80, 900 m), which is along the image ray originating from 0 m. Figure 11 shows the results from the case of symmetric surface aperture. We can see that the directionality of the focussed response is no longer defined by the solid blue line, but instead by the solid red line that denotes the image wavefront orientation at that location. The results for the asymmetric case are shown in Fig. 12, where the directionality of the focussed response is defined by the dashed red line, which combines the effects from asymmetric surface aperture and image‐ray bending. The observed results agree with those from the study of direct waves and the 1D model. We also note the observed artefacts, which are the results of the small surface aperture from −500 to 500 m and from −300 to 500 m used.

## 5 DISCUSSION

**Figure 13:**
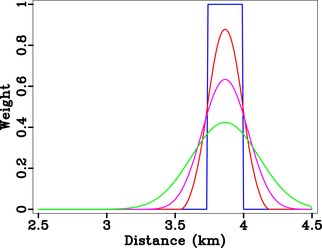
Various weight functions for data aperture used to study the compactness of the corresponding focussed responses.

**Figure 14:**
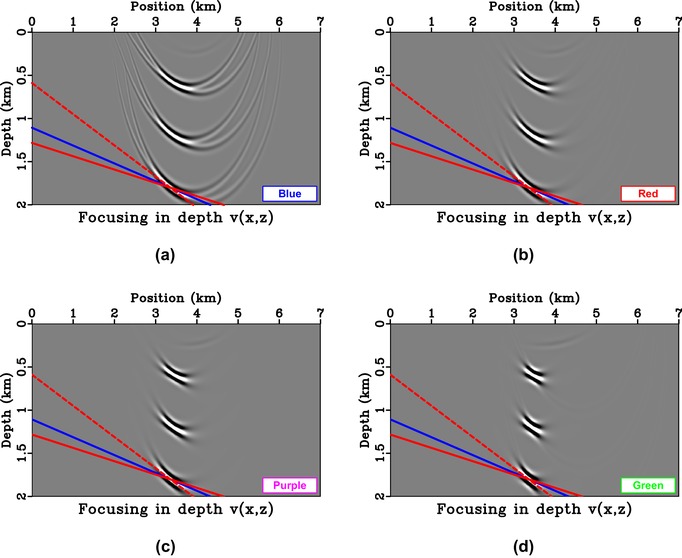
Direct‐wave focussing with asymmetric injection and various weighting schemes from Fig. 13; (a) Blue = Heaviside, (b) red, (c) magenta, and (d) green. One can observe a reduction in edge artefacts when a smoother weight is used but our conclusions regarding the radiating direction remain the same.

In our numerical examples, we limit the aperture of focussing functions by using a Heaviside weighting. Alternative methods with edge tapering may lead to smaller artefacts. Figures 13 and 14 show one such example, where we provide a comparison of back‐propagated direct‐wave focussed responses with smooth weights. We observe that the artefacts are tapered due to the smooth data weighting, but our observations regarding the effects of weighting on directionality still apply.

Moreover, we emphasize that limiting the aperture directly influences both compactness of the focussed response and performance of the Marchenko method. Unless a sufficient range of aperture is considered in the first place, the resulting focussing functions will contain artefacts from this truncation (van der Neut *et al*. 2015). As a consequence of this observation, it follows that for any fixed‐aperture data set, the directionality of the focussing functions depends not only on the medium properties, but on the relative position of the focal point to the source/receiver array.

**Figure 15:**
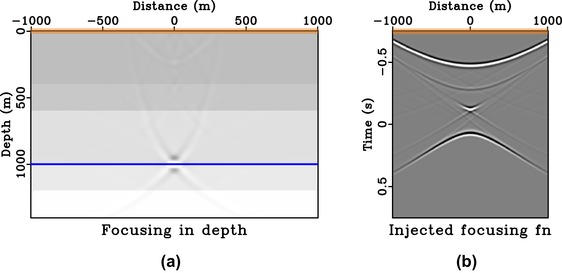
Focussing experiment in 1D model. Subpart (a) is the focussed response from injecting the focussing function shown in (b) that is obtained with a large symmetric aperture from −1000 to 1000 m. Note the reduced artefacts in (a), in comparison with Fig. 8.

**Figure 16:**
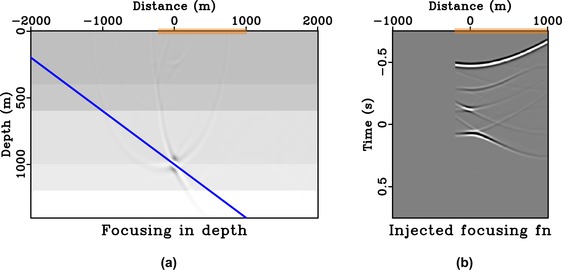
Focussing experiment in 1D model with asymmetric injection. Subpart (a) is the focussed response from injecting the focussing function shown in (b) that is obtained with a large but asymmetric aperture from −200 to 1000 m. The image rays are vertical in this example and the directionality of the focussed response is controlled by the effects of asymmetric injection (solid blue). Note the decrease in artefacts in comparison with Fig. 9.

In this study, we rely on straight‐ray geometry that is implicit in time‐domain imaging to draw our conclusions on the directionality of the focussed response. This assumption is exact for 1D media and approximately valid for small‐offset data in other (laterally heterogeneous) models. Therefore, there are noticeable artefacts in our injected panels (Figs. 8a, 9a, 11a, 12a) due to the use of short‐offset data. However, in a 1D model or media with a largely flat geology, a larger surface aperture can potentially be used to reduce artefacts. For example, in our Figs. 8(a) and  9(a), we can extend our experiments to a larger aperture from −1000 to 1000 m and from −200 to 1000 m, respectively. The results are shown in Figs. 15 and 16 with a notable decrease of artefacts, while our previous conclusions on the directionality of the focussed responses still hold.

In a companion paper (Sripanich *et al*. 2018), we further explore the Marchenko method in the context of time imaging. We show that the initial focussing function $f_{1,0}^{+}$ can be directly estimated from the local slopes of recorded CMP gathers, as opposed to using an explicit depth velocity model, which is generally less straightforward to obtain. The measured local slopes in the midpoint direction – associated with local subsurface structures – can be used together with the results from this study to create focussed responses (virtual sources) with special directionality such as orthogonal to the local subsurface structure in the time‐imaging domain.

Another possible implication of this study is to consider the focussing effects in the context of local (targeted) amplitude variation with offset (AVO) analysis. By utilizing the Marchenko method to eliminate the effects from the overburden, it is, in principle, possible to carry out an efficient time‐domain AVO analysis to estimate reflection coefficients free from overburden effects while honouring the finite‐aperture effects.

## 6 CONCLUSIONS

Building on the concept of time‐domain imaging, we provide further insight on directionality of focussed responses at depth from the injection of the Marchenko focussing functions. Assuming that the image rays are well defined with no caustics, the directional dependence of the focal pulse in Cartesian depth coordinates is a geometric superposition of the effects from limited aperture and the bending of image rays for time‐to‐depth conversion. This finding has implications to the construction of amplitude‐preserving redatuming and imaging, from focussing functions with controlled directionality and an appropriate handling of finite‐aperture effects.
